# Outcome of Patients Transplanted for C3 Glomerulopathy and Primary Immune Complex-Mediated Membranoproliferative Glomerulonephritis

**DOI:** 10.1016/j.ekir.2024.10.008

**Published:** 2024-10-15

**Authors:** Matthieu Halfon, Patrick Taffé, Christian Bucher, Fadi Haidar, Uyen Huynh-do, Laila-Yasmin Mani, Thomas Schachtner, Caroline Wehmeier, Jean-Pierre Venetz, Manuel Pascual, Fadi Fakhouri, Dela Golshayan, Swiss Transplant Cohort Study, Patrizia Amico, John-David Aubert, Vanessa Banz, Sonia Beckmann, Guido Beldi, Christoph Berger, Ekaterine Berishvili, Isabelle Binet, Pierre-Yves Bochud, Sanda Branca, Heiner Bucher, Emmanuelle Catana, Yves Chalandon, Sabina De Geest, Sophie De Seigneux Michael Dickenmann, Joëlle Lynn Dreifuss, Michel Duchosal, Thomas Fehr, Sylvie Ferrari-Lacraz, Christian Garzoni, Christophe Gaudet, Déla Golshayan, Nicolas Goossens, Jörg Halter, Dominik Heim, Christoph Hess, Sven Hillinger, Hans H. Hirsch, Patricia Hirt, Günther Hofbauer, Uyen Huynh-Do, Franz Immer, Michael Koller, Mirjam Laager, Bettina Laesser, Frédéric Lamoth, Roger Lehmann, Alexander Leichtle, Oriol Manuel, Hans-Peter Marti, Michele Martinelli, Valérie McLin, Katell Mellac, Aurelia Mercay, Karin Mettler, Nicolas J. Mueller, Antonia Müller, Ulrike Müller-Arndt, Beat Müllhaupt, Mirjam Nägeli, Graziano Oldani, Manuel Pascual, Jakob Passweg, Klara Posfay-Barbe, Juliane Rick, Anne Rosselet, Simona Rossi, Silvia Rothlin, Frank Ruschitzka, Thomas Schachtner, Urs Schanz, Stefan Schaub, Simon Schwab, Aurelia Schnyder, Macé Schuurmans, Thierry Sengstag, Federico Simonetta, Jürg Steiger, Guido Stirniman, Ueli Stürzinger, Christian Van Delden, Jean-Pierre Venetz, Jean Villard, Julien Vionnet, Madeleine Wick, Markus Wilhlem, Patrick Yerly

**Affiliations:** 1 Transplantation Center, Departments of Medicine and Surgery, Lausanne University Hospital and University of Lausanne, Switzerland; 2 Division of Biostatistics, University Center for Primary Care and Public Health, University of Lausanne, Lausanne, Switzerland; 3 Division of Nephrology and Transplantation Medicine, Kantonsspital St Gallen, St Gallen, Switzerland; 4 Department of Surgery, Service of Transplantation, Geneva University Hospitals, University of Geneva, Geneva, Switzerland; 5 Department of Nephrology and Hypertension, Inselspital University Hospital Bern, University of Bern, Bern, Switzerland; 6 Division of Nephrology, University Hospital Zürich, Zürich, Switzerland; 7 Clinic for Transplantation Immunology and Nephrology, University Hospital Basel, Basel, Switzerland; 8 Service of Nephrology and Hypertension, Lausanne University Hospital and University of Lausanne, Switzerland; 9 Transplantation Immunopathology Laboratory, Service of Immunology, Lausanne University Hospital and University of Lausanne, Switzerland

**Keywords:** C3 glomerulopathy, cohort study, complement pathway, graft outcome, kidney transplantation, membranoproliferative glomerulonephritis

## Abstract

**Introduction:**

Approximately 50% of patients with C3 glomerulopathy (C3G) and primary immune complex-mediated membranoproliferative glomerulonephritis (IC-MPGN) reach kidney failure 10 years after diagnosis. Because these patients are generally young, the majority will be listed for kidney transplantation (KTx). However, reported outcomes in patients transplanted for C3G and IC-MPGN are heterogeneous and conflicting, because they are mainly based on retrospective monocentric studies. We thus aimed to provide detailed multicenter data on these patients, taking advantage of the ongoing nationwide Swiss Transplant Cohort Study (STCS).

**Methods:**

We analyzed patient and graft outcomes, including the risk of graft loss in relation to recurrence of glomerulopathy.

**Results:**

Forty-one (10 C3G and 31 IC-MPGN) transplanted recipients were included with a mean age at transplantation of 48 ± 16 years. Living donors provided 53% of the organs. During a mean follow-up of 4.7 years, 7 patients (4 C3G and 3 IC-MPGN) presented disease recurrence with a mean time to recurrence of 1.2 years. New-onset or rapidly increasing proteinuria was an early marker of recurrence, preceding significant decline in estimated glomerular filtration rate (eGFR). Following recurrence, 28% lost their graft, compared to 11% of patients without recurrence. Disease recurrence was the primary cause of graft loss in all patients. Finally, 14% of patients died during follow-up.

**Conclusion:**

This study provides important insights into the epidemiology and outcome of patients with C3G and IC-MPGN and their grafts after KTx. The data also suggest that proteinuria may serve as an early biomarker of disease recurrence and should be considered in patient management as well as an endpoint in current clinical trials using novel complement modulators.

## Graphical abstract

**Figure:**
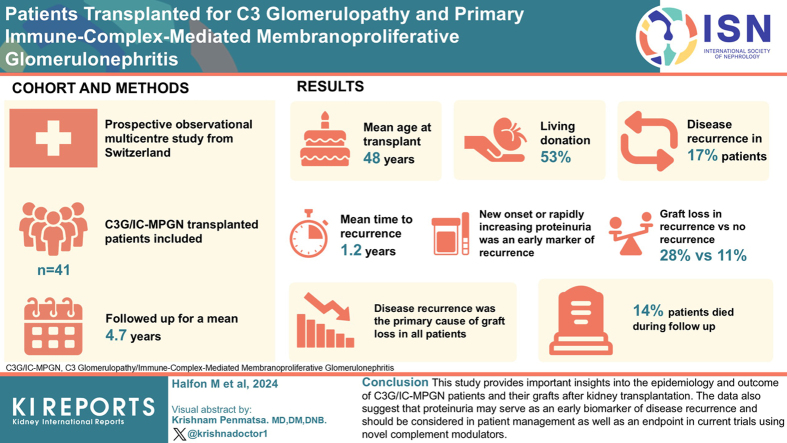

See Commentary on Page 7

C3G and idiopathic or primary IC-MPGN are rare glomerular diseases, characterized by glomerular deposition of complement, particularly C3, and/or immunoglobulins. These conditions arise from dysregulation of the complement alternative pathway, notably by a loss of control of the activity of the C3 convertase, leading to an overactivation of the alternative pathway.^1^^,^^2^ Experts are now viewing these entities as part of a spectrum of diseases sharing common pathophysiological mechanisms, rather than distinct disorders.^2^^,^^3^ Notably, both C3G and IC-MPGN may be associated to genetic or acquired complement abnormalities.^2^ C3G and IC-MPGN have a relatively low incidence, ranging from 0.5 to 1 case per 1 million patients per year, but have a dramatic renal prognosis.^4^ In fact, nearly 50% of patients diagnosed with C3G or IC-MPGN will progress to end-stage kidney disease (ESKD) within 10 years after the diagnosis.^4^^,^^5^ This highlights the impact of these diseases on kidney function and the need for effective management strategies. Indeed, current available drugs that target the complement system, such as complement factor C5 blockade, have failed to demonstrate significant efficacy in treating C3G or IC-MPGN in native and in transplanted kidneys.^3^^,^^6^, ^7^, ^8^ Due to the relatively young age of patients with C3G or IC-MPGN, the majority will be listed for a KTx.^7^^,^^9^ However, studies analyzing patient and graft outcomes in these transplant recipients report conflicting findings, because they are mainly retrospective monocentric reports or based on the older classification for membranoproliferative glomerulonephritis.^7^^,^^10^, ^11^, ^12^

With the rapid development of new promising drugs that target the alternative pathway of complement activation, it is crucial to provide more accurate and detailed data on the outcome of patients and grafts following KTx.^13^ Therefore, the aim of this study was to take advantage of the large nationwide STCS to assess the outcome of patients with C3G and primary IC-MPGN after KTx. We has analyzed disease recurrence and possible associations with acute rejection, graft dysfunction and loss, and patient survival.

## Methods

### Study Design and Participants

The study followed the principles of the Declaration of Helsinki and Istanbul and was approved by our Cantonal Ethics Committee (CER-VD, 2021-0173). This study (project number FUP172) is an observational study nested in the STCS, a prospective nationwide longitudinal cohort study in solid organ transplantation in Switzerland, approved by all the participating centers’ Ethics committees (2018–02394).^14^^,^^15^ The acceptance rate of participation in the STCS is >90% among all transplant recipients. All patients enrolled in the STCS have signed an informed consent and agreed to the prospective collection of clinical and biological data.

Our study included all STCS adult patients (aged ≥18 years) with a first KTx between May 2008 and December 2021 (*N* = 2631). Patients were excluded if they were aged <18 years, had previous KTx or multi-organ transplantation. Among the STCS kidney subcohort, 41 patients had an initial diagnosis of C3G or IC-MPGN as cause of ESKD. This diagnosis was based on biopsies of the native kidney together with the clinical evaluation by the referent nephrologist. Two patients had the concomitant diagnosis of monoclonal gammopathy, and no patient had active viral hepatitis or cryoglobulinemia.

### Data Collection

Within the STCS, patient and transplantation-specific data were collected at baseline (day 0 before transplantation surgery), then prospectively at months 6 and 12, and yearly using standardized case-report forms. The following baseline donor and recipient data were extracted for our study: sex, age, body mass index, type of donation (living or deceased donor, preemptive or not), cause of ESKD, type and duration of dialysis, cardiovascular comorbidities (hypertension, coronary heart disease, peripheral vascular disease, cerebrovascular disease), diabetes, and use of immunosuppressive drugs before KTx. Transplantation-related data further included: cold ischemia time, human leucocytes antigen mismatches, and immunosuppressive induction treatment. Follow-up data included maintenance immunosuppressive regimen, graft function (serum creatinine, μmol/l; proteinuria, g/d), graft loss (and cause of graft loss), patient’s death (and cause of death), recurrence of disease, and acute rejection episodes. All biopsies (per protocol and per cause) were recorded in the STCS database and scored by each center’s referent pathologist, according to the Banff 2009 and 2017 update classification.^16^^,^^17^

### Study Endpoints

The following outcomes were investigated: rejection episodes, disease recurrence, graft loss, graft dysfunction and death of the patient. Only biopsy-proven acute rejection episodes were analyzed, regrouping T cell and antibody-mediated rejection. We excluded biopsies with findings of “borderline changes,” and, per patient, considered only the first episode of biopsy-proven acute rejection for the analysis. Regarding the recurrence of disease, only biopsy-proven recurrence was considered, with all biopsy results confronted to the clinical data and confirmed by the nephrologist in charge of the patient. Graft function (eGFR) was calculated by the Chronic Kidney Disease Epidemiology Collaboration equation, and graft loss defined as return to dialysis or preemptive retransplantation. We also considered as additional outcome “graft dysfunction,” defined as the composite endpoint of death-censored graft loss, or eGFR < 30 ml/min per 1.73 m^2^, or proteinuria of more than 1 g/d, at different time-points of follow-up after KTx. Proteinuria was included in the composite endpoint because it has been shown to correlate with graft outcome.^18^, ^19^, ^20^

The primary endpoints were the cumulative incidence of graft dysfunction and graft loss during follow-up (up to 12 years post transplantation), according to the initial diagnosis of ESKD (C3G vs. IC-MPGN, as well as C3G and IC-MPGN vs. other causes) and to disease recurrence. The secondary endpoints were the occurrence of rejection episodes and patient survival during follow-up, according to the initial disease subgroup.

### Statistical Analyses

Data are presented as absolute numbers with percentages for categorical data, as means ± SD or medians and interquartile ranges (IQRs) for continuous variables. The comparison between patients transplanted for C3G and IC-MPGN, and the comparison between the group of C3G and IC-MPGN KTx recipients and patients transplanted for other causes, was performed using the chi-square test or Fisher exact test for categorical data and the *t* test or Mann-Whitney U test for continuous data. A *P*-value < 0.05 was considered statistically significant. The trend in the yearly number of patients transplanted was assessed using linear regression. Death and graft loss were the 2 main competing events and each of the following events: rejection, disease recurrence, and graft dysfunction were in turn treated as a third competing event in the analysis of the time to the occurrence of the first event. Cause-specific cumulative incidence functions (i.e., the probability curve of a specific event occurring over time) were nonparametrically estimated and the Gray nonparametric test was used to compare the cumulative incidence functions of the different subgroups of patients defined by their risk factors. All the analyses were carried out using Stata 17 statistical software.

## Results

### Study Population

Donor, recipient, and transplantation baseline characteristics are described in Table 1. Among the 2631 consecutive patients transplanted during the study period and included in the STCS, 701 (27%) were transplanted for glomerulonephritis (GN-KTx) and among them 41 (6%) had an initial diagnosis of C3G or IC-MPGN (10 C3G and 31 IC-MPGN). Compared to other recipients (Supplementary Table S1), patients transplanted for C3G or IC-MPGN (C3G/IC-MPGN-KTx) were younger at the time of transplantation (mean age 48 ± 16 years), with patients with C3G being significantly younger (mean age 34 ± 15 vs. 53 ± 13 years, for C3G and IC-MPGN KTx recipients, respectively; *P* = 0.01). As compared to the whole KTx cohort, patients with C3G and those with IC-MPGN predominantly received organs from living donors (50% and 55%, respectively) with a mean donor age that was slightly lower for C3G-KTx than for IC-MPGN-KTx (45 ± 20 years vs. 53 ± 13 years, respectively).

**Table 1:** Baseline donor, recipient, and transplantation characteristics

Variable	KTx for C3G/IC-MPGN (*n* = 41)	C3G-KTx (*n* = 10)	IC-MPGN-KTx (*n* = 31)	*P*-value
Recipient sex				
male/female (%)	25/16 (61/39)	6/4 (60/40)	19/12 (61/39)	0.9
Mean age at transplantation				
yrs ± SD	48 ± 16	34 ± 15	53 ± 13	0.01
History of coronary heart disease				
*n* (%)	6 (14)	1 (10)	5 (16)	0.6
History of cerebrovascular disease				
*n* (%)	0	0	0	1
History of peripheral vascular disease				
*n* (%)	3 (7)	0 (0)	3 (10)	0.3
History of hypertension				
*n* (%)	31 (77)	6 (60)	25 (80)	0.2
History of diabetes				
*n* (%)	1 (2)	0 (0)	1 (3)	0.6
Prior immunosuppression before KTx				
*n* (%)^a^	12 (29)	5 (50)	7 (23)	0.2
Preemptive transplantation				
*n* (%)	4 (10)	0 (0)	4 (13)	0.2
Type of donor				
living/deceased, *n* (%)	22/19 (53/47)	5/5 (50/50)	17/14 (55/45)	0.8
Mean donor age				
yrs ± SD	51 ± 15	45 ± 20	53 ± 13	0.1
Mean dialysis vintage				
yrs ± SD^b^ (*n*)	2.4 ± 2.9 (36)	1.2 ± 1.4 (10)	2.9 ± 3.2 (26)	0.1
Median HLA mismatches				
(IQR1–IQR3)	4 (3–5)	4.5 (3–5)	4 (3–5)	0.3
Induction therapy				
None, *n* (%)	2 (5)	0	2 (6)	0.7
Basiliximab, *n* (%)	32 (78)	9 (90)	23 (74)	
Anti-thymocyte globulin, *n* (%)	6 (15)	1 (10)	5 (16)	
Other, *n* (%)	1 (2)	0	1 (3)	
CNI-based maintenance immunosuppression, *n* (%)	41 (100)	10 (100)	31 (100)	0.9
Tacrolimus, *n* (%)	29 (70)	7 (70)	22 (71)	
Cyclosporine, *n* (%)	5 (12)	1 (10)	4 (13)	
Tacrolimus or cyclosporine alternatively, during follow-up, *n* (%)	7 (17)	2 (20)	5 (16)	

C3G, C3 glomerulopathy; CNI, calcineurin inhibitor; HLA, human leucocyte antigen; IC-MPGN, immune complex-mediated membranoproliferative glomerulonephritis, KTx, kidney transplantation.

Patients were stratified based on C3G or IC-MPGN. Chi-square, Fisher test or *t* test was used when appropriate.

a Missing data for 2 patients.

b Missing data for 1 patient.

Patients were given induction and maintenance immunosuppression according to their immunological risk status. For the whole cohort, the majority of C3G-KTx or IC-MPGN KTx recipients (78%) received basiliximab as induction therapy, antithymocyte globulin was used in 15% of the patients, 2 patients received rituximab in addition during the induction phase for an ABO-incompatible KTx, and 1 patient received intravenous immunoglobulins because of preformed donor-specific antibodies. There was no preemptive administration of eculizumab or other immunomodulatory drugs at the time of KTx in the patients with C3G or IC-MPGN. The standard maintenance immunosuppressive therapy was a combination of calcineurin inhibitors (CNI), prednisone at tapering doses, and mycophenolic acid-based agents (Table 1). Regarding CNI, a large preference was given to tacrolimus as compared to cyclosporine. During follow-up, 5 patients were switched to mammalian target of rapamycin inhibitors, 2 as a replacement of CNI and 3 instead of mycophenolic acid. This decision was mainly prompted to lower the burden of immunosuppression. Indeed, 1 patient presented with resistant cytomegalovirus infection, 2 others faced persistent BK viremia, and 1 patient was diagnosed with a nonmelanoma skin cancer. For the last patient, the switch was initiated following inclusion in a clinical trial. In 2 patients, this switch was only transient with restauration of the initial therapy after 1 year. Two patients were switched from CNI or mammalian target of rapamycin inhibitors to belatacept, one due to tacrolimus-induced thrombotic microangiopathy (confirmed by graft biopsy showing severe signs of CNI arteriolar toxicity and no deposition of immunoglobulin or C3) and the other because of mammalian target of rapamycin-associated side effects.

Fifty percent of patients with C3G and 23% of those with IC-MPGN had a history of immunosuppressive treatment before transplantation. However, this variable is not detailed in the STCS baseline case-report forms and we could not retrieve more precisions due to the retrospective nature of these data. A genetic workup was conducted to identify mutations in complement genes in a subset of 7 patients. Among these, only 3 patients (1 with IC-MPGN and 2 with C3G) were found to carry mutations in complement genes, more precisely presenting as heterozygotes for mutations in factor H-related protein (CFHR)1, heterozygotes for CFHR3, and deletions in the CFHR1 and CFHR3 genes.

We computed the yearly number of patients with C3G or IC-MPGN who underwent KTx in the STCS (expressed as number of transplanted patients, per 100 KTx per year) between 2008 and 2019. There was a trend toward increased KTx for ESKD due to C3G or IC-MPGN over the years, with higher numbers of transplanted patients during the 2013 to 2019 time-period, compared to 2008 to 2012 (*P* = 0.3) (Supplementary Figure S1).

### Graft Outcome

**Figure 1:**
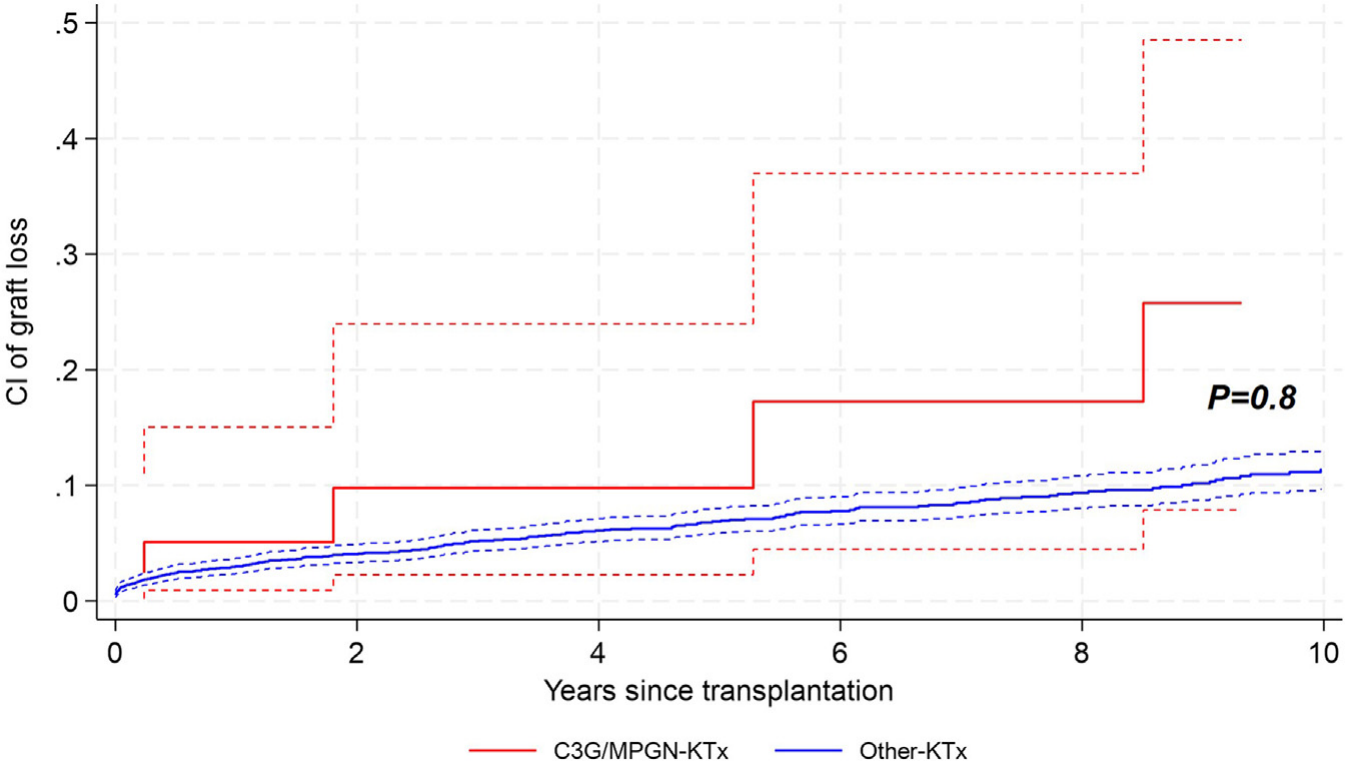
Cumulative incidence of graft loss over time, comparing 41 patients transplanted for C3 glomerulopathy and primary immune complex-mediated membranoproliferative glomerulonephritis and 2590 kidney transplant recipients transplanted for other causes. Full red line: cumulative incidence of graft loss for patients transplanted for C3 glomerulopathy and primary immune complex-mediated membranoproliferative glomerulonephritis (C3G/MPGN-KTx); dashed red lines: 95% confidence intervals. Full blue line: cumulative incidence of graft loss for patients transplanted for other causes (Other-KTx); dashed blue lines: 95% confidence intervals. CI, cumulative incidence; KTx, Kidney transplantation.

**Figure 2:**
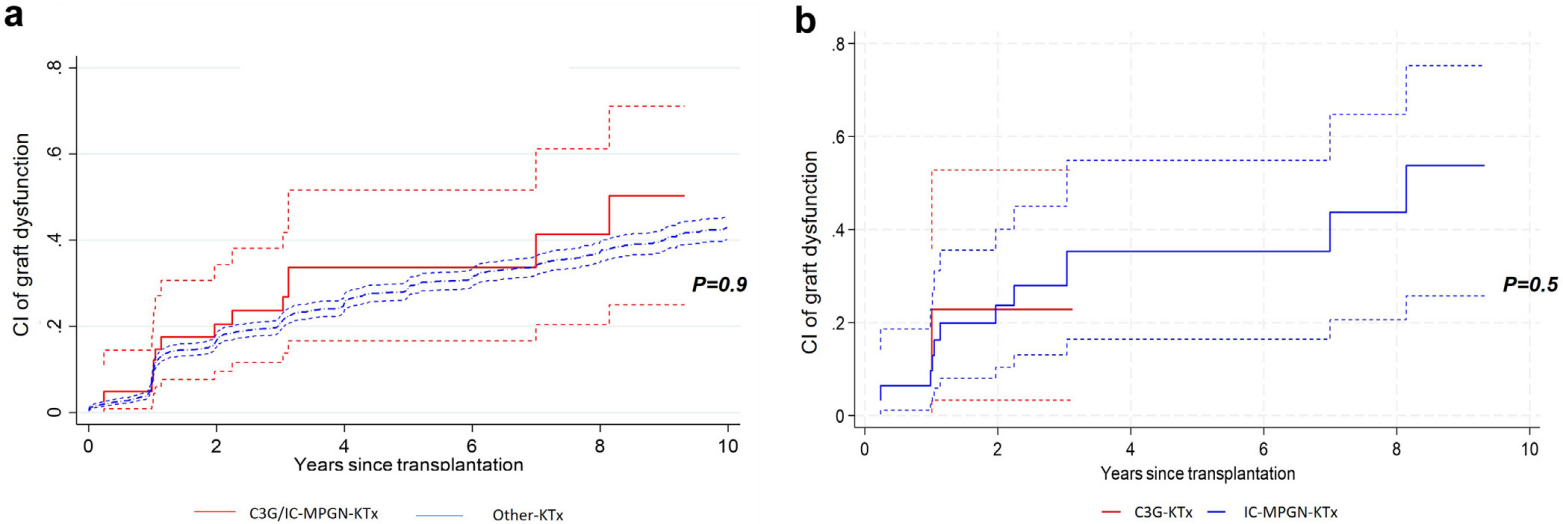
Cumulative incidence of graft dysfunction over time, comparing 41 patients transplanted for C3 glomerulopathy and primary immune complex-mediated membranoproliferative glomerulonephritis and 2590 kidney transplant recipients transplanted for other causes. (a) Comparison between the whole cohort and patients transplanted for C3 glomerulopathy and primary immune complex-mediated membranoproliferative glomerulonephritis. Full red line: cumulative incidence of graft dysfunction for patients transplanted for C3 glomerulopathy and primary immune complex-mediated membranoproliferative glomerulonephritis (C3G/MPGN-KTx); dashed red lines: 95% confidence intervals. Thick dashed blue line: cumulative incidence of graft dysfunction for patients transplanted for other causes (Other-KTx); thin dashed blue lines: 95% confidence intervals. (b) Comparison between patients transplanted for C3 glomerulopathy (*n* = 10) and patients transplanted for primary immune complex-mediated membranoproliferative glomerulonephritis (*n* = 31). Full red line: cumulative incidence of graft dysfunction for patients transplanted for C3 glomerulopathy (C3G-KTx); dashed red lines: 95% confidence intervals. Full blue line: cumulative incidence of graft dysfunction for patients transplanted for primary immune complex-mediated membranoproliferative glomerulonephritis (IC-MPGN-KTx); dashed blue lines: 95% confidence intervals. CI, cumulative incidence; KTx, kidney transplantation.

For C3G/IC-MPGN-KTx patients, during a mean follow-up of 4.7 years (IQR: 1.8–6.9), 6 patients (15%) lost their graft (mean time 4.9 years, IQR: 2.1–7.8); of these, 1 was a C3G and 5 were IC-MPGN KTx recipients. In addition, 14 patients (2 C3G and 12 IC-MPGN) (34%) reached the endpoint graft dysfunction in a mean time of 3.6 years (IQR: 1–7). In comparison, for patients transplanted for other causes, during a mean follow up of 5.3 years (IQR: 2.3–8.1), 206 patients (8%) lost their graft (mean time 3.0 years, IQR: 0.3–4.9) and 778 (30%) reached graft dysfunction (mean time 3.0 years, IQR: 1.0–4.1); thus, overall had a better graft outcome. The cumulative incidence of graft loss for C3G/IC-MPGN-KTx was 5.0% (95% confidence interval [CI]: 0–15.0), 9.7% (95% CI: 2.2–23.9), and 25.8% (95% CI: 7.9–48.5), as compared to 4.0% (95% CI: 3.3–4.9), 6.8% (95% CI: 5.8–8.0), and 10.3% (95% CI: 8.8–11.9) for patients transplanted for other causes at 2, 5, and 9 years, respectively (Figure 1). The cumulative incidence of graft dysfunction for C3G/IC-MPGN-KTx was 17.5% (95% CI: 7.6–30.7), 26.8% (95% CI: 13.9–41.7), and 50.3% (95% CI: 25.0–71.1), as compared to 16.7% (95% CI: 15.2–18.2), 28.8% (95% CI: 26.9–30.8), and 40.0% (95% CI: 38.0–43.2) for patients transplanted for other causes at 2, 5, and 9 years, respectively (Figure 2a). There was no difference in the occurrence of the endpoint graft dysfunction over time when comparing C3G to IC-MPGN KTx recipients directly (Figure 2b).

### Rejection Episodes

Nine patients (22%) of the C3G/IC-MPGN-KTx subgroup (2 C3G and 7 IC-MPGN) had at least 1 episode of acute rejection (within a mean time of 1.7 years, IQR: 0.04–0.8**)**. The types of rejection were 6 (66%) T cell-mediated rejection mainly during the first 6 months, 1 (11%) antibody-mediated rejection and 2 (33%) mixed cellular and antibody-mediated rejection. All rejection episodes were managed by methylprednisolone pulses and an overall increase in maintenance immunosuppression; and 1 patient received a combination therapy of corticosteroids, antithymocyte globulin, and plasma exchanges. For patients transplanted for other causes, 691 (27%) presented with at least 1 episode of acute rejection (within a mean time of 0.7 years, IQR: 0.03–0.8), with 58% T cell-mediated rejection, 14% antibody-mediated rejection and 28% mixed rejection episodes. Compared to patients transplanted for other causes, the cumulative incidence of rejection was not significantly different (*P* = 0.26) (Supplementary Figure S2A). Similarly, there was no significant difference (*P* = 0.85) between C3G-KTx and IC-MPGN-KTx regarding the cumulative incidence of rejection (Supplementary Figure S2B).

### Disease Recurrence

During follow-up, 7 patients (17%) (4 with the initial diagnosis of C3G and 3 with IC-MPGN) presented a recurrence of disease within a mean time of 1.2 years (IQR: 0.4–1.7; no significant difference in time to recurrence between both subgroups). Except for 1 patient, all biopsies with a final diagnosis of disease recurrence were clinically indicated. Increase of serum creatinine was the cause of biopsy for 2 patients and new-onset significant proteinuria for 4 patients. Among the 7 patients experiencing recurrence, 6 exhibited microhematuria. Upon recurrence, the median serum creatinine level stood at 115 μmol/l (IQR: 99–132), and the median recorded proteinuria was 2.0 g/d (IQR: 0.97–2.5). Serum complement levels were not systematically assessed during follow-up and at the time of disease recurrence. Nevertheless, we could retrieve serum C3 values in 6 of these patients, and notably, 3 of them exhibited low C3 levels at the time of recurrence. Furthermore, soluble C5b-9 levels were analyzed in the serum of 4 patients, revealing elevated levels in 3 individuals and normal level in 1, at the time of recurrence. Regarding the therapeutic management of disease recurrence, 1 patient received plasma exchange followed by maintenance therapy with eculizumab, 2 patients received prolonged maintenance therapy with eculizumab, 1 was treated with rituximab, and 1 was treated with an increased dose of oral prednisone (1 mg/kg/d) for 3 months after recurrence. The remaining patients received no specific therapy. A comprehensive description of pre transplantation and post transplantation characteristics and the subsequent evolution of individual patients with recurrence of C3G and IC-MPGN is provided in Supplementary Table S2.

**Figure 3:**
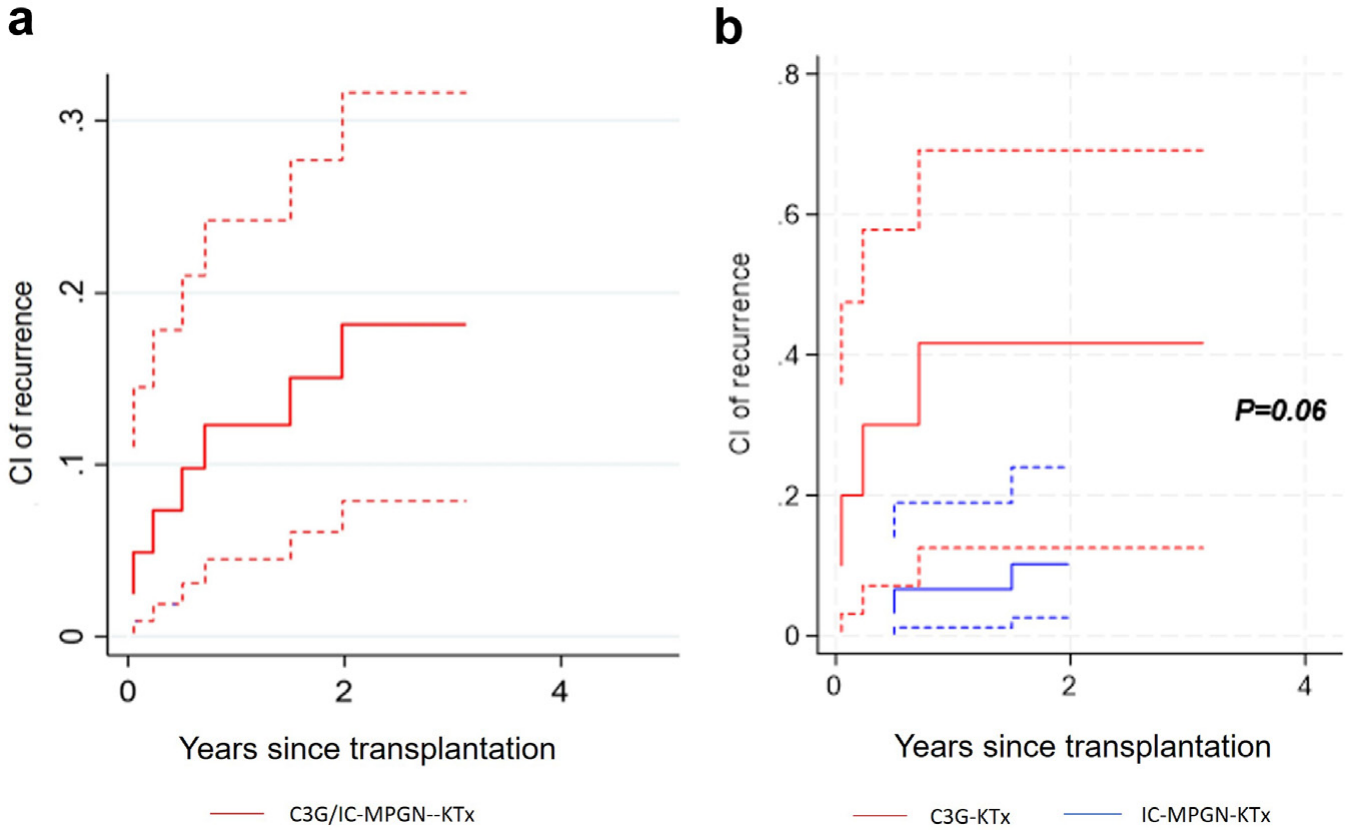
(a) Cumulative incidence of recurrence of disease over time, among 10 patients transplanted for C3 glomerulopathy and 31 patients transplanted for primary immune complex-mediated membranoproliferative glomerulonephritis. (b) Full red line: cumulative incidence of recurrence for patients transplanted for C3 glomerulopathy (C3G-KTx); dashed red lines: 95% confidence intervals. Full blue line: cumulative incidence of recurrence for patients transplanted for primary immune complex-mediated membranoproliferative glomerulonephritis (IC-MPGN-KTx); dashed blue lines: 95% confidence intervals. KTx, kidney transplantation.

**Figure 4:**
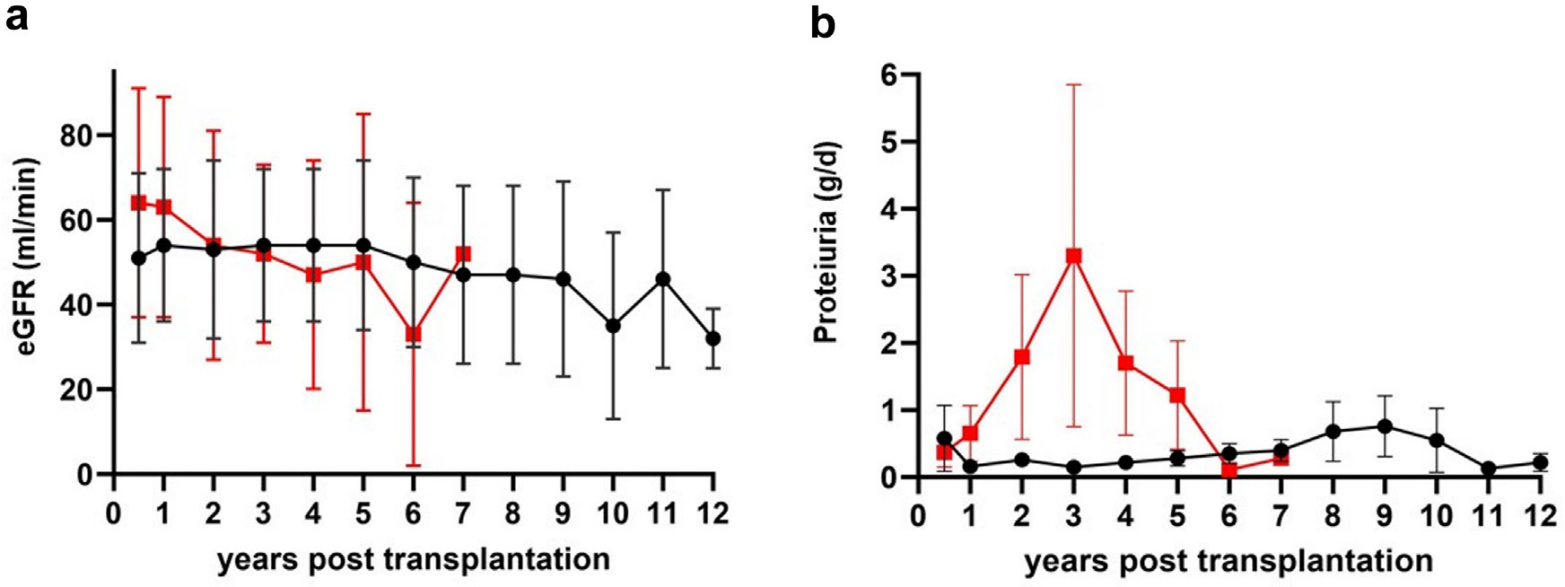
Evolution of (a) kidney function and (b) proteinuria during follow-up, comparing recurrent and nonrecurrent C3G/IC-MPGN kidney transplant recipients. Red squares and line: recurrent group; black dots and line: non-recurrent group. C3G, C3 glomerulopathy; IC-MPGN, immune complex-mediated membranoproliferative glomerulonephritis.

**Figure 5:**
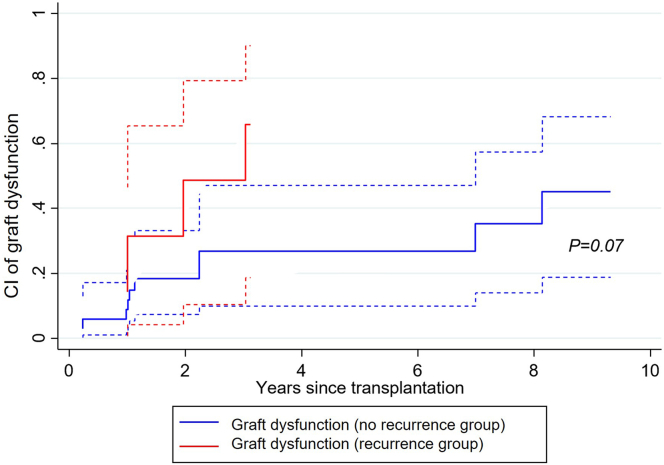
Cumulative incidence of graft dysfunction among 41 patients with recurrent and non-recurrent C3G/IC-MPGN after kidney transplantation. Full red line: cumulative incidence of graft dysfunction for the recurrent group; dashed red lines: 95% confidence intervals. Full blue line: cumulative incidence of graft dysfunction for the non-recurrent group; dashed blue lines: 95% confidence intervals. C3G, C3 glomerulopathy; CI, cumulative incidence; IC-MPGN, immune complex-mediated membranoproliferative glomerulonephritis.

The overall cumulative incidence of C3G/IC-MPGN recurrence after KTx and between C3G-KTx and IC-MPGN-KTx in our cohort is depicted in Figure 3. C3G-KTx had a trend toward a higher risk of recurrence compared to IC-MPGN-KTx (*P* = 0.06). When compared to the nonrecurrent group, patients with recurrent C3G/IC-MPGN after KTx were younger (mean age at transplantation 40 ± 21 vs. 50 ± 14 years; in recurrent and nonrecurrent C3G/IC-MPGN, respectively; *P* = 0.1), were twice more likely to have received immunosuppressive therapy before transplantation (57% vs. 23%; *P* = 0.2), and, none had benefitted from preemptive KTx (0% vs. 12%; *P* = 0.3) (Table 2). The progression of eGFR and 24-hour proteinuria throughout the follow-up period between recurrent and nonrecurrent C3G/IC-MPGN KTx recipients is represented in Figure 4. Interestingly, proteinuria was an early marker of recurrence, preceding significant decline in eGFR. During the follow-up, 2 patients (28%) with recurrent C3G/IC-MPGN lost their graft compared to 4 (11%) that had no recurrence (mean time to graft loss 4.1 years, IQR: 3.6–4.7; and 4.9 years, IQR: 1.4–8.7, respectively). Among the 34 patients without recurrence, 13 experienced a notable increase in serum creatinine or proteinuria during follow-up. Eleven of these patients underwent kidney biopsies, revealing acute rejection in 7 cases, thrombotic microangiopathy secondary to CNI toxicity in 1 patient (i.e., there was no mesangial hypercellularity or endocapillary proliferation, no deposition of immunoglobulin or C3, with exclusively arteriolar lesions displaying fibrin deposits and severe hyalinosis), chronic allograft nephropathy in 2, and acute tubular necrosis in 1 patient. For the remaining patients without biopsies, graft pyelonephritis and acute tubular necrosis were identified as the causes of graft dysfunction. Regarding patients with recurrent C3G/IC-MPGN, recurrence was the primary cause of graft loss with a mean time between recurrence and graft loss of 3.8 years (IQR: 3.1–4.4). Moreover, 4 patients (57%) with recurrent C3G/IC-MPGN reached graft dysfunction during follow-up (mean time 2.3 years, IQR: 1.7–3.1) compared to 10 patients (29%) with nonrecurrent C3G/IC-MPGN (mean time 4.2 years, IQR: 1.0–7.8). There was thus a trend toward a higher risk to reach the outcome graft dysfunction during follow-up for patients with recurrent C3G/IC-MPGN compared to nonrecurrent C3G/IC-MPGN (cumulative risk 66%, 95% CI: 18.7–90.0 vs. 45%, 95% CI: 18.9–68.2; *P* = 0.07) (Figure 5). For recurrent C3G/IC-MPGN, the mean time between recurrence and graft dysfunction was 0.8 years (IQR: 0–1.2).

**Table 2:** Baseline donor, recipient, and transplantation characteristics in non-recurrent and recurrent C3 glomerulopathy and primary immune complex-mediated membranoproliferative glomerulonephritis

Variable	Nonrecurrent C3G (*n* = 6)	Recurrent C3G (*n* = 4)	Nonrecurrent IC-MPGN (*n* = 28)	Recurrent IC-MPGN (*n* = 3)
Recipient sex				
male/female (%)	4/2 (66/33)	2/2 (50/50)	18/10 (64/36)	1/2 (33/66)
Mean age at transplantation				
yrs ± SD	38 ± 16	29 ± 14	53 ± 12	56 ± 21
History of coronary heart disease				
*n* (%)	1 (17)	0	5 (18)	0
History of cerebrovascular disease				
*n* (%)	0	0	0	0
History of peripheral vascular disease				
*n* (%)	0	0	3 (11)	0
History of hypertension				
*n* (%)	3 (50)	3 (75)	22 (78)	3 (100)
History of diabetes				
*n* (%)	0	0	1 (3)	0
Prior immunosuppression before KTx				
*n* (%)^a^	2 (33)	3 (75)	6 (21)	1 (33)
Preemptive transplantation				
*n* (%)	0	0	4 (14)	0
Type of donor				
living/deceased, *n* (%)	3/3 (50/50)	2/2 (50/50)	15/13 (53/47)	2/1 (66/33)
Mean donor age				
yrs ± SD	48 ± 21	39 ± 19	53 ± 12	56 ± 21
Mean dialysis vintage				
yrs ± SD^b^ (*n*)	1.5 ± 1.8 (6)	0.75 ± 0.5 (4)	3 ± 3.3 (24)	2 ± 3 (2)
Median HLA mismatches				
(IQR1–IQR3)	4 (3–5)	4 (3–5)	4 (3–5)	2 (1–4)
Induction therapy				
None, *n* (%)	0	0	2 (6)	0
Basiliximab, *n* (%)	5 (83)	4 (100)	20 (71)	3 (100)
Anti-thymocyte globulin, *n* (%)	1 (17)	0	5 (18)	0
Other, *n* (%)	0	0	1 (3)	0
CNI-based maintenance immunosuppression, *n* (%)	6 (100)	4 (100)	28 (100)	3 (100)
Tacrolimus, *n* (%)	4 (66)	3 (75)	19 (68)	3 (100)
Cyclosporine, *n* (%)	1 (17)	0	4 (14)	0
Tacrolimus or cyclosporine alternatively during follow-up, *n* (%)	1 (17)	1 (25)	5 (18)	0

C3G, C3 glomerulopathy; CNI, calcineurin inhibitor; HLA, human leucocyte antigen; IC-MPGN, immune complex-mediated membranoproliferative glomerulonephritis, KTx, kidney transplantation.

Patients were stratified based on recurrence or non-recurrence of C3G/IC-MPGN. Fisher test or *t*-test was used when appropriate.

a Missing data for 2 patients.

b Missing data for 1 patient.

### Patient Outcome

**Figure 6:**
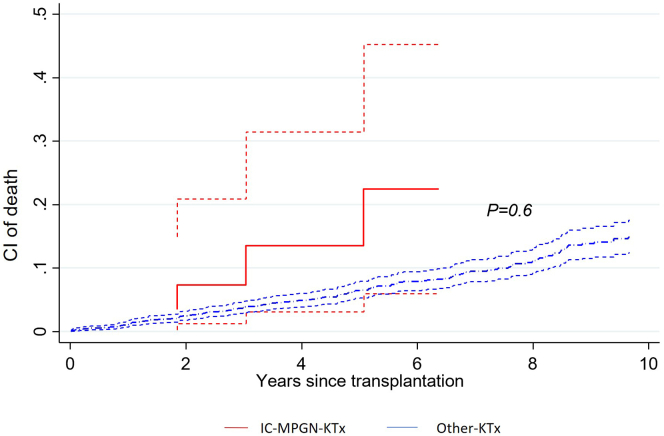
Cumulative incidence of death during follow-up, between 31 patients transplanted for primary immune complex-mediated membranoproliferative glomerulonephritis and 2590 kidney transplant recipients transplanted for other causes. Full red line: cumulative incidence of death for patients transplanted for primary immune complex-mediated membranoproliferative glomerulonephritis (IC-MPGN-KTx); dashed red lines: 95% confidence intervals. Thick dashed blue line: cumulative incidence of death for patients transplanted for other causes **(**Other-KTx); thin dashed blue lines: 95% confidence intervals. C3G, C3 glomerulopathy; CI, cumulative incidence; IC-MPGN, immune complex-mediated membranoproliferative glomerulonephritis; KTx, kidney transplantation.

Six of 41 patients (14%) died (mean time 4.6 years, IQR: 2.2–7.3), all from the IC-MPGN-KTx group. Five of the 6 patients (83%) who died had reached graft dysfunction before dying. The cumulative incidence of death for IC-MPGN-KTx patients was 2.7% (95% CI: 0–12.4), 10.5% (95% CI: 2.5–25.2), and 22.9% (95% CI: 7.6–43.1); at 2, 5, and 7 years, respectively (Figure 6). The causes of death were infections (*n* = 2), oncological complications (1 patient with posttransplant lymphoproliferative disease), neurodegenerative disorder (*n* = 1), sudden death (*n* = 1) and 1 patient after palliative care following graft loss. Of patients transplanted for other causes, 294 (11%) died during follow-up (mean time 4.4 years, IQR: 1.5–6.9). The cumulative incidence of death for patients transplanted for other cause was 2.9% (95% CI: 2.3–3.6), 6.8 (95% CI: 5.7–8.1) and 9.7% (95% CI: 8.3–11.2); at 2, 5, and 7 years, respectively. Cancer-related deaths (13%), infections (15%), and cardiovascular diseases (17%) were the most common causes of death in this group.

## Discussion

Using a large observational multicenter KTx cohort, we analyzed the epidemiology of C3G and IC-MPGN as well as patient and graft outcome after transplantation. Although a rarer cause of ESKD, there was a trend toward a higher number of KTx for C3G and IC-MPGN over the years in the STCS. We observed that the overall long-term patient and graft prognosis was significantly impacted by the recurrence of disease.

The STCS dataset revealed a trend toward an increased incidence of KTx for C3G and IC-MPGN in the past decade. This could indicate a better recognition and improved diagnosis by clinicians, attributed to the new 2010 classification for MPGN.^21^ However, the worldwide prevalence of C3G/IC-MPGN remains very low. For instance, in a large cohort of native kidney biopsies from India, the overall prevalence of C3G was only 1.52%. Similarly, in a multicenter registry of biopsy-proven glomerulonephritis in Japan, the combined prevalence of C3G and IC-MPGN accounted for only 1.3% of total kidney biopsies.^22^^,^^23^ Interestingly, C3G/IC-MPGN-KTx constituted 6% of all KTx for glomerulonephritis cases in our cohort, indicating an overrepresentation of these diseases in KTx patients, as compared to the population with chronic kidney disease. This disparity not only emphasizes the unfavorable prognosis of C3/IC-MPGN in native kidneys due to the absence of validated and efficacious drugs, but also raises the possibility of increased access to transplantation for patients with C3G or IC-MPGN. Indeed, this subgroup of patients is overall younger and with less comorbidities compared to patients transplanted for other causes and may therefore have a greater likelihood to be considered eligible candidates for KTx.

The rate of graft loss during follow-up observed in our study (15%) was lower than previously reported (ranging from 17% to 50%).^8^^,^^11^^,^^12^^,^^24^ However, it should be noted that some studies classified patients using the old MPGN classification or used a composite endpoint to define graft loss, which is more comparable to our rate of “graft dysfunction” at 34%.^8^^,^^12^^,^^25^ Nonetheless, the percentage of graft loss in C3G/IC-MPGN-KTx patients was higher than that observed in other KTx recipients within the STCS during the same follow-up period. Notably, this higher rate of graft loss occurred without a significant increase in rejection episodes compared to patients transplanted for other causes, suggesting that recurrence may be the primary factor contributing to this difference in prognosis.^26^

In comparison to other studies, the recurrence rate in the STCS was significantly lower. Previous reports have indicated recurrence rates ranging from 23% to 84%.^8^^,^^11^^,^^12^^,^^24^ However, it is important to note that some studies utilized early protocol biopsies to diagnose recurrence, whereas in our cohort, except for 1 patient, all recurrences were diagnosed by clinically indicated biopsies. In addition, our report includes only 2 patients with monoclonal gammopathy and it is known that IC-MPGN-KTx patients with monoclonal gammopathy have a higher risk of recurrence (odds ratio 5.6; *P* = 0.01) and also experience a shorter time to recurrence with overall worse prognosis.^11^^,^^24^^,^^27^^,^^28^ Those differences could explain the disparity between our study and previous reports. Nevertheless, our findings underscore the significant impact of recurrence on graft outcome. Clinically, recurrence primarily manifested as an increase or new-onset significant proteinuria without a notable elevation in serum creatinine levels in most of the cases initially. For our patients, rigorous urine monitoring protocols during follow-up and the level of proteinuria allowed early diagnosis of recurrence, more consistently than hematuria or changes in serum creatinine. This parameter could thus be considered as a biomarker for disease recurrence and activity during follow-up or as a surrogate of outcome and an endpoint in future interventional therapeutic trials with novel complement-targeted drugs.^29^ Importantly, C3G and IC-MPGN KTx patients with disease recurrence had a trend toward worse prognosis and were nearly 3 times more likely to lose their grafts.

Currently, there are no approved therapies specifically targeting recurrent C3G or IC-MPGN after KTx. Given the study period (2008–2021), treatment protocols at recurrence were heterogenous among centers, and only few patients were put on complement-targeted therapy preventively or at the time of recurrence. Indeed, only 3 of the 7 patients with recurrence were treated with eculizumab, with mixed results (Supplementary Table S2). This is comparable with previous studies where eculizumab failed to show consistent positive results in the treatment of recurrent C3G or IC-MPGN.^8^^,^^29^ Therefore, there is a critical need for drugs that specifically target C3 activation. There are high expectations for drugs that are currently tested in phase 2 and 3 clinical trials such as pegcetacoplan (polyethylene glycol-conjugated peptide that binds to C3 and C3b, inhibiting the C3 convertase and downstream activation) and iptacopan (factor B inhibitor).^30^^,^^31^ They could dramatically change the prognosis of recurrent C3G and IC-MPGN after KTx and future trials will be needed to assess the long-term efficacy and safety of these therapies.

Only a few studies have presented data on the survival of C3G and IC-MPGN-KTx patients. Moroni *et al.*^12^ reported a death rate of 16% for IC-MPGN-KTx, whereas Kumar *et al.*^10^ reported a death rate of 19% for C3G-KTx in a retrospective monocentric study; both are higher than in our cohort. Although the study of Kumar *et al.*^10^ may not be a proper comparison because it was obtained in an Indian cohort (50% of deaths due to graft failure and financial inability to obtain drugs and adequate follow-up), Moroni *et al.*^12^ report varied causes of death such as sepsis, cancer, myocardial infarction, graft loss, pancreatis, and gastric hemorrhage. In our multicenter national cohort, the cumulative incidence of death was higher in IC-MPGN-KTx (no deaths in the C3G subgroup) as compared to the overall cohort of kidney transplant recipients. As previously reported, the causes of death in our cohort were diverse. Interestingly, 5 of 6 patients who died during follow-up after KTx had also reached the composite endpoint of graft dysfunction (a composite of death-censored graft loss, eGFR < 30 ml/min per 1.73 m^2^, or proteinuria of more than 1 g/d) before their death. It is well-established that both low eGFR and proteinuria are associated with increased morbidity and mortality in chronic kidney disease. Therefore, graft dysfunction could have contributed to the high mortality rate observed in our IC-MPGN-KTx cohort.^32^ Thus, by preserving graft function, we could potentially decrease mortality in this subgroup of relatively young patients.

The strength of our study lies in its multicenter design nested within the STCS. We also used for analysis, a cumulative incidence risk model (i.e., a competing risk model) which is more appropriate than the conventional (and biased) Kaplan-Meier analysis published in previous reports. However, the STCS is a general observational KTx cohort, not specifically dedicated to the follow-up of C3G/IC-MPGN-KTx patients. As a result, specific data, such as detailed pretransplant data, exhaustive genetic analyses, or systematic measurements of serum complement at clinically relevant timepoints, are either unavailable or had to be obtained retrospectively. Comprehensive data on these aspects could have provided valuable insights into the underlying mechanisms and potential predictors of recurrence and outcome in this subset of KTx patients. Nevertheless, the inclusion of >90% of KTx patients in the STCS allows us to report on practically all cases of C3G and IC-MPGN transplanted and followed in Switzerland since 2008. This comprehensive coverage enhances the reliability of our findings, albeit in a relatively small subset of patients. Another limitation could be the lack of protocol biopsies at defined time-points after KTx. Indeed, the STCS is an observational prospective cohort and the decision for kidney graft biopsy is made based on the center’s protocol and referent nephrologist (4 of the 6 Swiss KTx centers perform protocol biopsies at 3 or 6 months and then at 1 year after KTx). However, regarding the subset of patients with the initial diagnosis of C3G or primary IC-MPGN, all centers performed a biopsy at month 3 or 6 as a baseline, and in the case of proteinuria appearance or rising creatinine during follow-up.

### Conclusion

To the best of our knowledge, our study represents the first nationwide cohort of C3G and primary IC-MPGN KTx recipients. Our findings demonstrate that these patients have a slightly worse outcome compared to other KTx patients. However, the recurrence of the disease significantly worsens the prognosis and may indirectly contribute to overall mortality by exacerbating graft dysfunction. We also observed that proteinuria can serve as an early marker of disease recurrence. Overall, the data highlight the importance of effective management strategies to prevent disease recurrence and improve the long-term outcome for C3G and IC-MPGN KTx recipients.

## Appendix

### List of the Swiss Transplant Cohort Study

Patrizia Amico, John-David Aubert, Vanessa Banz, Sonia Beckmann, Guido Beldi, Christoph Berger, Ekaterine Berishvili, Isabelle Binet, Pierre-Yves Bochud, Sanda Branca, Heiner Bucher, Emmanuelle Catana, Yves Chalandon, Sabina De Geest, Sophie De Seigneux Michael Dickenmann, Joëlle Lynn Dreifuss, Michel Duchosal, Thomas Fehr, Sylvie Ferrari-Lacraz, Christian Garzoni, Christophe Gaudet, Déla Golshayan, Nicolas Goossens, Jörg Halter, Dominik Heim, Christoph Hess, Sven Hillinger, Hans H. Hirsch, Patricia Hirt, Günther Hofbauer, Uyen Huynh-Do, Franz Immer, Michael Koller (Head, Data center), Mirjam Laager, Bettina Laesser, Frédéric Lamoth, Roger Lehmann, Alexander Leichtle, Oriol Manuel, Hans-Peter Marti, Michele Martinelli, Valérie McLin, Katell Mellac, Aurelia Mercay, Karin Mettler, Nicolas J. Mueller (Chairman Scientific Committee), Antonia Müller, Ulrike Müller-Arndt, Beat Müllhaupt, Mirjam Nägeli, Graziano Oldani, Manuel Pascual (Executive office), Jakob Passweg, Klara Posfay-Barbe, Juliane Rick, Anne Rosselet, Simona Rossi, Silvia Rothlin, Frank Ruschitzka, Thomas Schachtner, Urs Schanz, Stefan Schaub, Simon Schwab, Aurelia Schnyder, Macé Schuurmans, Thierry Sengstag, Federico Simonetta, Jürg Steiger (Head, Executive office), Guido Stirniman, Ueli Stürzinger, Christian Van Delden (Executive office), Jean-Pierre Venetz, Jean Villard, Julien Vionnet, Madeleine Wick (STCS coordinator), Markus Wilhlem, and Patrick Yerly.

## Disclosure

MH has obtained funding from the Swiss Kidney Foundation and the Fondation Lausannoise pour la Transplantation d’Organes (FLTO). DG is supported by the Fondation Medi-CAL Futur and the Faculty of Biology and Medicine of the University of Lausanne. All the other authors declared no competing interests.

## Supplementary Material

Supplementary File (PDF) **Figure S1.** Yearly number of patients transplanted for end-stage kidney disease due to C3 glomerulopathy and primary immune complex-mediated membranoproliferative glomerulonephritis in the study population. **Figure S2.** Cumulative incidence of acute rejection episodes over time. Comparison between: (A) 41 patients transplanted for C3 glomerulopathy and primary immune complex-mediated membranoproliferative glomerulonephritis and 2590 kidney transplant recipients transplanted for other causes, and (B) 10 patients transplanted for C3 glomerulopathy and 31 patients transplanted for primary immune complex-mediated membranoproliferative glomerulonephritis. **Table S1.** Baseline donor, recipient, and transplantation characteristics of patients transplanted for other causes than C3 glomerulopathy (C3G) and primary immune complex-mediated membranoproliferative glomerulonephritis (IC-MPGN). **Table S2.** Detailed pretransplantation and posttransplantation characteristics of patients with recurrent C3 glomerulopathy and primary immune complex-mediated membranoproliferative glomerulonephritis. STROBE Checklist.

## Footnotes

Supplementary File (PDF)
**Figure S1.** Yearly number of patients transplanted for end-stage kidney disease due to C3 glomerulopathy and primary immune complex-mediated membranoproliferative glomerulonephritis in the study population. **Figure S2.** Cumulative incidence of acute rejection episodes over time. Comparison between: (A) 41 patients transplanted for C3 glomerulopathy and primary immune complex-mediated membranoproliferative glomerulonephritis and 2590 kidney transplant recipients transplanted for other causes, and (B) 10 patients transplanted for C3 glomerulopathy and 31 patients transplanted for primary immune complex-mediated membranoproliferative glomerulonephritis. **Table S1.** Baseline donor, recipient, and transplantation characteristics of patients transplanted for other causes than C3 glomerulopathy (C3G) and primary immune complex-mediated membranoproliferative glomerulonephritis (IC-MPGN). **Table S2.** Detailed pretransplantation and posttransplantation characteristics of patients with recurrent C3 glomerulopathy and primary immune complex-mediated membranoproliferative glomerulonephritis. STROBE Checklist.

## References

[1] Caravaca-Fontan F., Praga M. (2022). Prognostication for C3 glomerulopathy and idiopathic immunoglobulin-associated membranoproliferative glomerulonephritis. Clin J Am Soc Nephrol.

[2] Noris M., Daina E., Remuzzi G. (2021). Membranoproliferative glomerulonephritis: no longer the same disease and may need very different treatment. Nephrol Dial Transplant.

[3] Fakhouri F., Le Quintrec M., Fremeaux-Bacchi V. (2020). Practical management of C3 glomerulopathy and Ig-mediated MPGN: facts and uncertainties. Kidney Int.

[4] Lomax-Browne H.J., Medjeral-Thomas N.R., Barbour S.J. (2022). Association of histologic parameters with outcome in C3 glomerulopathy and idiopathic immunoglobulin-associated membranoproliferative glomerulonephritis. Clin J Am Soc Nephrol.

[5] Fakhouri F., Schwotzer N., Golshayan D., Fremeaux-Bacchi V. (2022). The rational use of complement inhibitors in kidney diseases. Kidney Int Rep.

[6] Ruggenenti P., Daina E., Gennarini A. (2019). C5 convertase blockade in membranoproliferative glomerulonephritis: a single-arm clinical trial. Am J Kidney Dis.

[7] Bomback A.S., Smith R.J., Barile G.R. (2012). Eculizumab for dense deposit disease and C3 glomerulonephritis. Clin J Am Soc Nephrol.

[8] Regunathan-Shenk R., Avasare R.S., Ahn W. (2019). Kidney transplantation in C3 glomerulopathy: A case series. Am J Kidney Dis.

[9] Iatropoulos P., Daina E., Curreri M. (2018). Cluster analysis identifies distinct pathogenetic patterns in C3 glomerulopathies/immune complex-mediated membranoproliferative GN. J Am Soc Nephrol.

[10] Kumar A., Ramachandran R., Rawat A. (2021). Poor allograft outcome in Indian patients with post-transplant C3 glomerulopathy. Clin Kidney J.

[11] Lorenz E.C., Sethi S., Leung N., Dispenzieri A., Fervenza F.C., Cosio F.G. (2010). Recurrent membranoproliferative glomerulonephritis after kidney transplantation. Kidney Int.

[12] Moroni G., Casati C., Quaglini S. (2011). Membranoproliferative glomerulonephritis type I in renal transplantation patients: a single-center study of a cohort of 68 renal transplants followed up for 11 years. Transplantation.

[13] Hoy S.M. (2021). Pegcetacoplan: first approval. Drugs.

[14] Koller M.T., van Delden C., Muller N.J. (2013). Design and methodology of the Swiss Transplant Cohort Study (STCS): a comprehensive prospective nationwide long-term follow-up cohort. Eur J Epidemiol.

[15] Stampf S., Mueller N.J., van Delden C. (2021). Cohort profile: the Swiss Transplant Cohort Study (STCS): a nationwide longitudinal cohort study of all solid organ recipients in Switzerland. BMJ Open.

[16] Sis B., Mengel M., Haas M. (2010). Banff ’09 meeting report: antibody mediated graft deterioration and implementation of Banff working groups. Am J Transplant Off J Am Soc Transplant Am Soc Transpl Surg.

[17] Haas M., Loupy A., Lefaucheur C. (2018). The Banff 2017 Kidney Meeting Report: revised diagnostic criteria for chronic active T cell-mediated rejection, antibody-mediated rejection, and prospects for integrative endpoints for next-generation clinical trials. Am J Transplant.

[18] Fernandez-Fresnedo G., Plaza J.J., Sanchez-Plumed J., Sanz-Guajardo A., Palomar-Fontanet R., Arias M. (2004). Proteinuria: a new marker of long-term graft and patient survival in kidney transplantation. Nephrol Dial Transplant.

[19] Ponticelli C., Graziani G. (2012). Proteinuria after kidney transplantation. Transpl Int.

[20] Diena D., Messina M., De Biase C. (2019). Relationship between early proteinuria and long term outcome of kidney transplanted patients from different decades of donor age. BMC Nephrol.

[21] Sethi S., Fervenza F.C. (2012). Membranoproliferative glomerulonephritis-a new look at an old entity. N Engl J Med.

[22] Kumar A., Nada R., Ramachandran R. (2020). Outcome of C3 glomerulopathy patients: largest single-centre experience from South Asia. J Nephrol.

[23] Nakagawa N., Mizuno M., Kato S. (2021). Demographic, clinical characteristics and treatment outcomes of immune-complex membranoproliferative glomerulonephritis and C3 glomerulonephritis in Japan: a retrospective analysis of data from the Japan Renal Biopsy Registry. PLoS One.

[24] Zand L., Lorenz E.C., Cosio F.G. (2014). Clinical findings, pathology, and outcomes of C3GN after kidney transplantation. J Am Soc Nephrol.

[25] Angelo J.R., Bell C.S., Braun M.C. (2011). Allograft failure in kidney transplant recipients with membranoproliferative glomerulonephritis. Am J Kidney Dis.

[26] Golshayan D., Wojtowicz A., Bibert S. (2016). Polymorphisms in the lectin pathway of complement activation influence the incidence of acute rejection and graft outcome after kidney transplantation. Kidney Int.

[27] Caravaca-Fontan F., Polanco N., Villacorta B. (2022). Recurrence of immune complex and complement-mediated membranoproliferative glomerulonephritis in kidney transplantation. Nephrol Dial Transplant.

[28] Alasfar S., Carter-Monroe N., Rosenberg A.Z., Montgomery R.A., Alachkar N. (2016). Membranoproliferative glomerulonephritis recurrence after kidney transplantation: using the new classification. BMC Nephrol.

[29] Golshayan D., Schwotzer N., Fakhouri F., Zuber J. (2023). Targeting the complement pathway in kidney transplantation. J Am Soc Nephrol.

[30] Apellis (2022). Study assessing the safety and efficacy of pegcetacoplan in post-transplant recurrence of C3G or IC-MPGN (NOBLE) (NCT04572854). Clinicaltrialgov.

[31] Study on Efficacy and Safety of LNP023 in C3 glomerulopathy Patients Transplanted and not transplanted. https://ctv.veeva.com/study/study-on-efficacy-and-safety-of-lnp023-in-c3-glomerulopathy-patients-transplanted-and-not-transplant.

[32] Levin A., Stevens P.E. (2014). Summary of KDIGO 2012 CKD Guideline: behind the scenes, need for guidance, and a framework for moving forward. Kidney Int.

