# Supplementary material for: Outcome of Patients Transplanted for C3 Glomerulopathy and Primary Immune Complex-Mediated Membranoproliferative Glomerulonephritis
Source: Kidney Int Rep. 2024 Oct 15;10(1):75–86. doi: 10.1016/j.ekir.2024.10.008 (PMC11725970; doi:10.1016/j.ekir.2024.10.008)
Supplement: Supplementary File (PDF) — Figure S1. Yearly number of patients transplanted for end-stage kidney disease due to C3 glomerulopathy and primary immune complex-mediated membranoproliferative glomerulonephritis in the study population. Figure S2. Cumulative incidence of acute rejection episodes over time. Comparison between: (A) 41 patients transplanted for C3 glomerulopathy and primary immune complex-mediated membranoproliferative glomerulonephritis and 2590 kidney transplant recipients transplanted for other causes, and (B) 10 patients transplanted for C3 glomerulopathy and 31 patients transplanted for primary immune complex-mediated membranoproliferative glomerulonephritis. Table S1. Baseline donor, recipient, and transplantation characteristics of patients transplanted for other causes than C3 glomerulopathy (C3G) and primary immune complex-mediated membranoproliferative glomerulonephritis (IC-MPGN). Table S2. Detailed pretransplantation and posttransplantation characteristics of patients with recurrent C3 glomerulopathy and primary immune complex-mediated membranoproliferative glomerulonephritis. STROBE Checklist. [file mmc1.pdf]

Supplemental Material

Supplemental Table S1. Baseline donor, recipient, and transplantation characteristics of patients transplanted for other causes than C3 glomerulopathy (C3G) and primary immune-complex-mediated membranoproliferative glomerulonephritis (IC-MPGN).

Variable	***Other-KTx (n=2590)***
Recipient sex	
male/female (%)	1661/929 (64/36%)
Mean age at transplantation	
years ± SD	53 ± 13
Cause of ESKD, n (%)	
Diabetic nephropathy	268 (10%)
ADPKD	547 (21%)
Other causes	1115 (44%)
Glomerulonephritis (other than C3G/IC-MPGN)	660 (25%)
IgA nephropathy	323 (49%)
Focal segmental glomerulosclerosis	117 (18%)
GN-other	220 (33%)
History of coronary heart disease	
n (%)	484 (19%)
History of cerebrovascular disease	
n (%)	210 (8%)

History of peripheral vascular disease<br>n (%)	318 (12%)
History of hypertension<br>n (%)	2142 (83%)
History of diabetes<br>n (%)	422 (16%)
Prior immunosuppression before KTx<br>n (%)[a]	478 (18%)
Preemptive KTx<br>n (%)	475 (18%)
Type of donor<br>living/deceased, n (%)	1088/1502 (42/58%)
Mean donor age<br>years $\pm$ SD	53 $\pm$ 16
Mean dialysis vintage<br>years $\pm$ SD[b] (n)	2.6 $\pm$ 2.5 (1950)
Median HLA mismatches[c]<br>(IQR1-IQR3)	4 (1-5)
Induction therapy<br>None<br>Basiliximab<br>Anti-thymocyte globulin<br>Other	 120 (5%)<br>1716 (66%)<br>600 (23%)<br>154 (6%)
CNI-based maintenance immunosuppression (n, %)<br>Tacrolimus only	2578 (99%)<br>1931 (74%)

Cyclosporine only	327 (3%)
Tacrolimus or cyclosporine during follow-up	21 (12%)

ADPKD: autosomal dominant polycystic kidney disease, C3G: C3 glomerulopathy, CNI: calcineurin inhibitor, ESKD: end-stage kidney disease, GN: glomerulonephritis, HLA: human leucocyte antigen, IC-MPGN: primary immune-complex-mediated membranoproliferative glomerulonephritis, KTx: kidney transplantation, other-KTx: transplantation for other causes than C3G and IC-MPGN.

[a] data missing for 106 patients.
[b] data missing for 165 patients.
[c] data missing for 6 patients.

Supplemental Table S2. Detailed pre- and post-transplantation characteristics of patients with recurrent C3 glomerulopathy and primary immune-complex-mediated membranoproliferative glomerulonephritis.

Data	Patient 1	Patient 2	Patient 3	Patient 4	Patient 5	Patient 6	Patient 7
**Primary diagnosis**	IC-MPGN	IC-MPGN	IC-MPGN	C3G	C3G	C3G	C3G
**Sex**	Male	Female	Female	Female	Female	Female	Male
**Age at diagnosis (years)**	26	64	35	11	12	5	46
**Initial clinical presentation**	NA	Progressive renal failure with nephrotic range proteinuria	NA	NA	Retinopathy. Nephrotic syndrome.	NA	NA
**Genetic workup**	Not done	Not done	Not done	Not done	No mutation found	CFHR1 mutation	Not done
**Serologic workup before KTx**	NA	No monoclonal gammopathy. Normal C3 and C4.	Negative C3Nef. Presence of monoclonal gammopathy.	Negative C3NeF. No monoclonal gammopathy.	Negative C3NeF. No monoclonal gammopathy. Low C3, normal C4, elevated sC5b-9, elevated factor Bb.	Positive C3NeF.	No monoclonal gammopathy. Elevated factor Bb.
**Time to ESKD (years)**	NA	13	17	6.5	15	12.5	1.7
**Age at KTx (years)**	34	77	56	19	29	18	49
**Dialysis type**	HD	HD	HD	PD	PD	HD	HD
**Donor type**	living unrelated	living related	brain dead donor	brain dead donor	living related	brain dead donor	living related
**Induction regimen**	basiliximab	basiliximab	basiliximab	basiliximab	basiliximab	basiliximab	basiliximab
**Maintenance regimen**	Tac-MMF	Tac-MMF	Tac-MMF	Tac-MMF	Tac-MMF	Tac-MMF	Tac-MMF
**Time to recurrence (months)**	18	5	24	37	9	0.5	3
**Clinical presentation at recurrence**	sCreat 115 µmol/l, proteinuria 300 mg/d, glomerular hematuria.	sCreat 135 µmol/l, proteinuria 1.6 g/d, glomerular hematuria.	sCreat 130 µmol/l, proteinuria 2 g/d, glomerular hematuria,	sCreat 86 µmol/l, proteinuria 2 g/d.	sCreat 216 µmol/l (baseline post KTx at 160 µmol/l), proteinuria 500 mg/d,	sCreat 80 µmol/l, proteinuria 3 g/d, glomerular hematuria.	sCreat 113 µmol/l, proteinuria 400 mg/d, glomerular hematuria.

					glomerular hematuria,		
**Serologic workup at recurrence**	Normal C3 and C4 at recurrence.	Normal C3 and C4 at recurrence.	Low C3 and normal C4 at recurrence.<br>Elevated factor Bb and sC5b-9 at recurrence, then low C3 during follow-up.	Low C3 and C4 at recurrence.<br>Elevated sC5b-9 at recurrence, then low C3 during follow-up.	Normal C3 and C4 at recurrence.<br>Normal sC5b-9 at recurrence, then low C3 during follow-up.	Low C3 and elevated sC5b-9 at recurrence, then low C3 during follow-up.	Elevated factor Bb at KTx and at recurrence.
**Recurrence biopsy**	*Optic:*<br>Mesangial hypercellularity<br><br>*IF:*<br>C3 ++ (membranous)<br>IgA +++ (membranous)<br>IgM ++ (membranous)<br>Kappa +++ (membranous)<br>Lambda +++ (membranous)	*Optic:*<br>Mesangial hypercellularity, endocapillary proliferation<br><br>*IF:*<br>C3 +++ (mesangial and membranous)<br>IgG ++ (mesangial and membranous)<br>Kappa monoclonal	*Optic:*<br>Mesangial hypercellularity, endocapillary proliferation<br><br>*IF:*<br>C3 +++ (mesangial)<br>IgG +++ (mesangial)<br>C1q (+) (mesangial)	*Optic:*<br>Diffuse mesangial hypercellularity, endocapillary proliferation<br><br>*IF:*<br>C3 +++ (mesangial and membranous)<br>IgM + (mesangial and membranous)	*Optic:*<br>Mesangial sclerosis<br><br>*IF:*<br>C3 ++ (mesangial)<br>sC5b-9 + (mesangial)	*Optic*:<br>Normal glomerulus<br><br>*IF:*<br>C3 +++ (membranous)<br>IgG + (membranous)<br>IgM + (membranous))	*Optic:*<br>Mesangial hypercellularity, endocapillary proliferation<br><br>*IF:*<br>C3 ++ (mesangial)<br>IgG + (mesangial)
**Recurrence treatment**	Conservative management	Conservative management	Prednisone high dose	Eculizumab	Eculizumab	Plasma exchange and eculizumab	Rituximab and prednisone
**Outcome**	Stable graft function with minor proteinuria.<br>No rejection episode.	Progressive deterioration of graft function with apparition of nephrotic range proteinuria.<br>Graft loss, 0.5 years after recurrence.<br>No rejection episode.	Stable graft function with regression of proteinuria.<br>No rejection episode.	Stable graft function with regression of proteinuria.<br>No rejection episode.	Progressive deterioration of graft function with apparition of nephrotic range proteinuria.<br>Graft loss, 6 years after recurrence (extended follow-up).<br>No rejection episode.	No rejection episode.	Progressive deterioration of graft function with apparition of nephrotic range proteinuria.<br>Graft loss, 5.5 years after recurrence.<br>No rejection episode.

C3G: C3 glomerulopathy, ESKD: end-stage kidney disease, HD: hemodialysis, IF: immunofluorescence, KTx: kidney transplantation, MMF: mycophenolate mofetil, MPGN: membrano-proliferative glomerulonephritis, NA: not available, PD: peritoneal dialysis, sCreat: serum creatinine, TAC: tacrolimus.

Supplemental Figure S1. Yearly number of patients transplanted for end-stage kidney disease due to C3G/IC-MPGN in the study population.

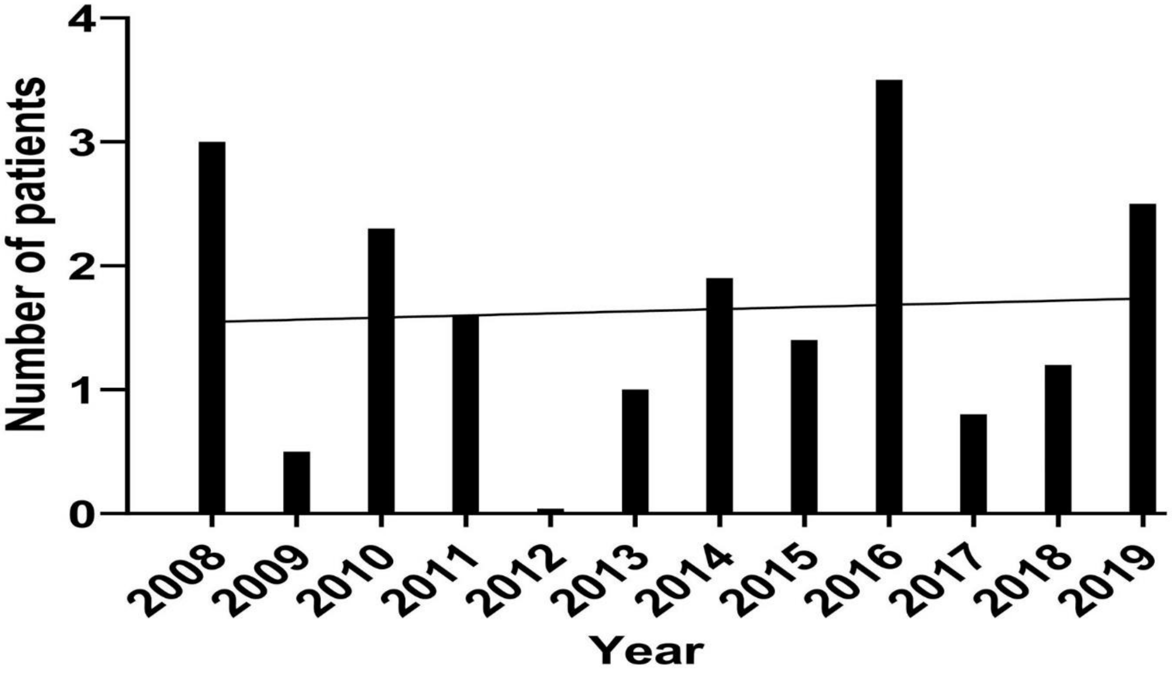

Black line: regression line.

The number of patients is expressed per 100 kidney transplantations per year. (*P*=0.3).

Supplemental Figure S2. Cumulative incidence of acute rejection episodes over time.

Comparison between: (**A**) 41 patients transplanted for C3 glomerulopathy and primary immune-complex-mediated membranoproliferative glomerulonephritis and 2590 kidney transplant recipients transplanted for other causes, and (**B**) 10 patients transplanted for C3 glomerulopathy and 31 patients transplanted for primary immune-complex-mediated membranoproliferative glomerulonephritis.

A. Full red line: cumulative incidence of rejection for patients transplanted for C3 glomerulopathy and primary immune-complex-mediated membranoproliferative glomerulonephritis (C3G/MPGN-KTx); dashed red lines: 95% confidence intervals. Thick dashed blue line: cumulative incidence of rejection for patients transplanted for other nephropathies (Other-KTx), thin dashed blue lines: 95% confidence intervals.
CI: cumulative incidence
B. Full red line: cumulative incidence of rejection for patients transplanted for C3 glomerulopathy (C3G-KTx); dashed red lines: 95% confidence intervals. Full blue line: cumulative incidence of rejection for patients transplanted for primary immune-complex-mediated membranoproliferative glomerulonephritis (IC-MPGN-KTx); dashed blue lines: 95% confidence intervals.

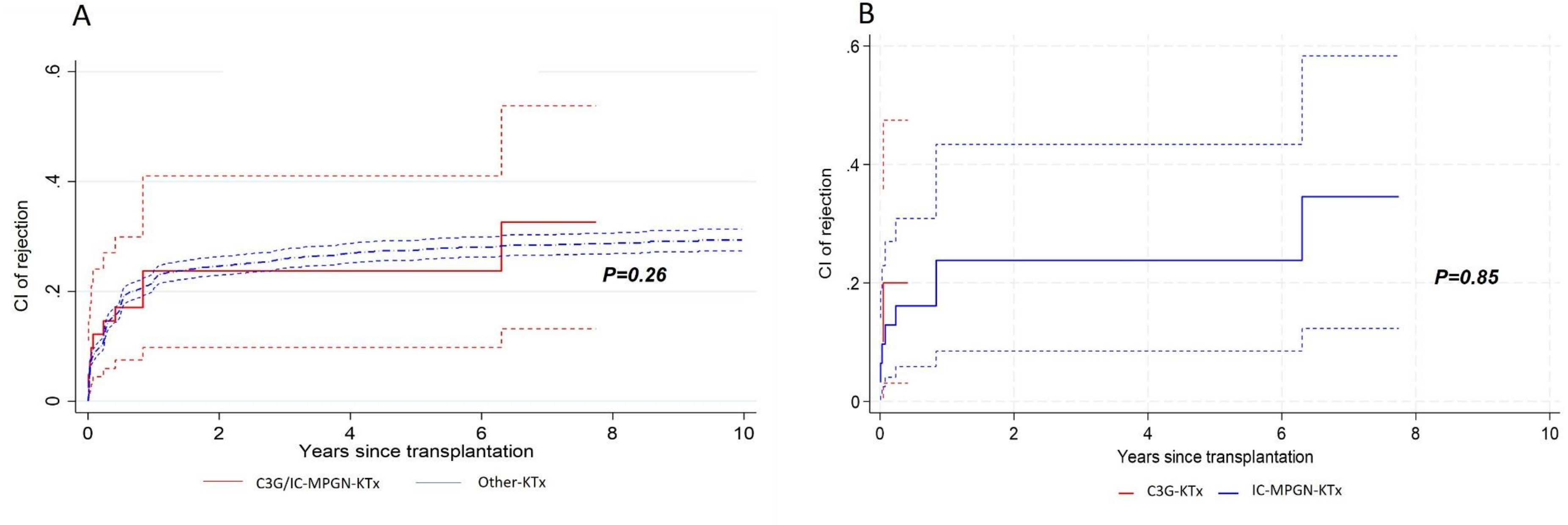

STROBE Statement—Checklist of items that should be included in reports of ***cohort studies***

	Item No	Recommendation	Page No
**Title and abstract**	1	(*a*) Indicate the study's design with a commonly used term in the title or the abstract	1
		(*b*) Provide in the abstract an informative and balanced summary of what was done and what was found	3
**Introduction**			
Background/rationale	2	Explain the scientific background and rationale for the investigation being reported	4
Objectives	3	State specific objectives, including any prespecified hypotheses	4
**Methods**			
Study design	4	Present key elements of study design early in the paper	5-7
Setting	5	Describe the setting, locations, and relevant dates, including periods of recruitment, exposure, follow-up, and data collection	5
Participants	6	(*a*) Give the eligibility criteria, and the sources and methods of selection of participants. Describe methods of follow-up	5-6
		(*b*) For matched studies, give matching criteria and number of exposed and unexposed	
Variables	7	Clearly define all outcomes, exposures, predictors, potential confounders, and effect modifiers. Give diagnostic criteria, if applicable	6

Data sources/ measurement	8*	For each variable of interest, give sources of data and details of methods of assessment (measurement). Describe comparability of assessment methods if there is more than one group	6
Bias	9	Describe any efforts to address potential sources of bias	N.A
Study size	10	Explain how the study size was arrived at	5
Quantitative variables	11	Explain how quantitative variables were handled in the analyses. If applicable, describe which groupings were chosen and why	6
Statistical methods	12	(*a*) Describe all statistical methods, including those used to control for confounding	6
		(*b*) Describe any methods used to examine subgroups and interactions	6
		(*c*) Explain how missing data were addressed	
		(*d*) If applicable, explain how loss to follow-up was addressed	6
		(*e*) Describe any sensitivity analyses	
**Results**			
Participants	13*	(a) Report numbers of individuals at each stage of study—eg numbers potentially eligible, examined for eligibility, confirmed eligible, included in the study, completing follow-up, and analysed	7
		(b) Give reasons for non-participation at each stage	N.A
		(c) Consider use of a flow diagram	
Descriptive data	14*	(a) Give characteristics of study participants (eg demographic, clinical, social) and information on exposures and potential confounders	7/20-21
		(b) Indicate number of participants with missing data for each variable of interest	21
		(c) Summarise follow-up time (eg, average and total amount)	8

Outcome data	15*	Report numbers of outcome events or summary measures over time	8-11
Main results	16	(*a*) Give unadjusted estimates and, if applicable, confounder-adjusted estimates and their precision (eg, 95% confidence interval). Make clear which confounders were adjusted for and why they were included	8-11
		(*b*) Report category boundaries when continuous variables were categorized	8-11
		(*c*) If relevant, consider translating estimates of relative risk into absolute risk for a meaningful time period	
Other analyses	17	Report other analyses done—eg analyses of subgroups and interactions, and sensitivity analyses	8-11
**Discussion**			
Key results	18	Summarise key results with reference to study objectives	11
Limitations	19	Discuss limitations of the study, taking into account sources of potential bias or imprecision. Discuss both direction and magnitude of any potential bias	13
Interpretation	20	Give a cautious overall interpretation of results considering objectives, limitations, multiplicity of analyses, results from similar studies, and other relevant evidence	11-13
Generalisability	21	Discuss the generalisability (external validity) of the study results	14
**Other information**			
Funding	22	Give the source of funding and the role of the funders for the present study and, if applicable, for the original study on which the present article is based	15

*Give information separately for exposed and unexposed groups.

Note: An Explanation and Elaboration article discusses each checklist item and gives methodological background and published examples of transparent reporting. The STROBE checklist is best used in conjunction with this article (freely available on the Web sites of PLoS Medicine at http://www.plosmedicine.org/, Annals of Internal Medicine at http://www.annals.org/, and Epidemiology at http://www.epidem.com/). Information on the STROBE Initiative is available at http://www.strobe-statement.org.
